# Shorter heels are linked with greater elastic energy storage in the Achilles tendon

**DOI:** 10.1038/s41598-021-88774-8

**Published:** 2021-04-30

**Authors:** A. D. Foster, B. Block, F. Capobianco, J. T. Peabody, N. A. Puleo, A. Vegas, J. W. Young

**Affiliations:** 1 Department of Anatomy, School of Osteopathic Medicine, Campbell University, PO Box 4280, Buies Creek, NC 27506 USA; 2 School of Osteopathic Medicine, Campbell University, PO Box 4280, Buies Creek, NC 27506 USA; 3 Department of Anatomy and Neurobiology, Northeast Ohio Medical University (NEOMED), Rootstown, OH 44272 USA

**Keywords:** Physiology, Anatomy

## Abstract

Previous research suggests that the moment arm of the *m.* *triceps surae* tendon (i.e., Achilles tendon), is positively correlated with the energetic cost of running. This relationship is derived from a model which predicts that shorter ankle moment arms place larger loads on the Achilles tendon, which should result in a greater amount of elastic energy storage and return. However, previous research has not empirically tested this assumed relationship. We test this hypothesis using an inverse dynamics approach in human subjects (n = 24) at speeds ranging from walking to sprinting. The spring function of the Achilles tendon was evaluated using specific net work, a metric of mechanical energy production versus absorption at a limb joint. We also combined kinematic and morphological data to directly estimate tendon stress and elastic energy storage. We find that moment arm length significantly determines the spring-like behavior of the Achilles tendon, as well as estimates of mass-specific tendon stress and elastic energy storage at running and sprinting speeds. Our results provide support for the relationship between short Achilles tendon moment arms and increased elastic energy storage, providing an empirical mechanical rationale for previous studies demonstrating a relationship between calcaneal length and running economy. We also demonstrate that speed and kinematics moderate tendon performance, suggesting a complex relationship between lower limb geometry and foot strike pattern.

## Introduction

The role of the Achilles tendon (AT) in elastic energy storage with subsequent return during stance phase is well established^1–7^. Recovery of elastic energy imparted to the AT is potentially influenced by AT morphology in three ways: (1) material properties of the tendon, (2) cross-sectional area of the tendon, and (3) the moment arm of the calcaneal tuberosity loading the tendon against the muscle force of the *m. triceps surae* (i.e., foot geometry). Previous work suggests that foot geometry may explain variation in how much potential energy is stored in the tendon, finding that a shorter AT moment arm is correlated with lower mass-specific energy costs of locomotion (COL; L O_2_ kg^−1^ s^−1^)^8, 9^. This finding suggests that shorter AT moment arms are associated with greater elastic loads imparted to the tendon, which are then recovered as kinetic energy during the support phase of each gait cycle^9^, thereby reducing COL. Scholz et al.^9^ also suggest that the length of the AT moment arm is a more significant factor in explaining COL than variation in material properties of the tendon itself or size-related variation in the cost of swinging the leg forward during the aerial phase of the gait cycle^9^. However, assumptions about the interacting roles of AT moment arm length, tendon cross-sectional dimensions, and tendon material properties on variation in elastic energy storage have yet to tested in an integrated manner. Moreover, Scholz et al.^9^ doesn’t directly measure the variables in the model which predict variation in elastic energy storage, including muscle force and the external moment arm. Finally, because previous studies of how AT moment arm length influences COL have used trained runners running on a treadmill at a speed of 16 km/h, it is still unknown how variation in speed and athletic training impacts elastic loading to the tendon in relation to moment arm length. While previous work has explored elastic loading of the AT at different speeds and under different loading conditions^10–21^, this study is the first to investigate the potential correlation between foot geometry like the AT moment arm length and spring-like behavior of this tendon in humans.

In this study, we model elastic loading of the AT by characterizing the spring-like behavior over the support phase of each gait cycle using two metrics. First, we calculate specific net work (SNW) at the ankle joint. SNW is a ratio of net joint work to total joint work that results in values from 0, comparable to a perfect spring, to 1, comparable to a perfect motor or brake (see “Methods” section)^22^. SNW values of 1.0 are the result of a step with solely positive or negative work at the joint during stance phase, whereas a value of 0 indicates commensurate levels of positive and negative work, consistent with spring-like behavior. We use SNW to test whether AT moment arm length predicts spring-like behavior of the tendon. Second, we also measure individual AT cross-sectional area and tendon length using ultrasonography along with the muscle force impulse at the ankle over stance phase to estimate the magnitude of energy storage in the AT^23^ (see “Methods” section). We test the following hypotheses:

H_1_: Subjects with shorter AT moment arms should exhibit more spring-like behavior (lower SNW values) in running gaits.

H_2_: AT moment arm length should be negatively correlated with tendon stress (i.e., force per unit cross-sectional area).

H_3_: AT moment arm length should be negatively correlated with tendon energy storage.

## Results

### Specific net work

**Figure 1:**
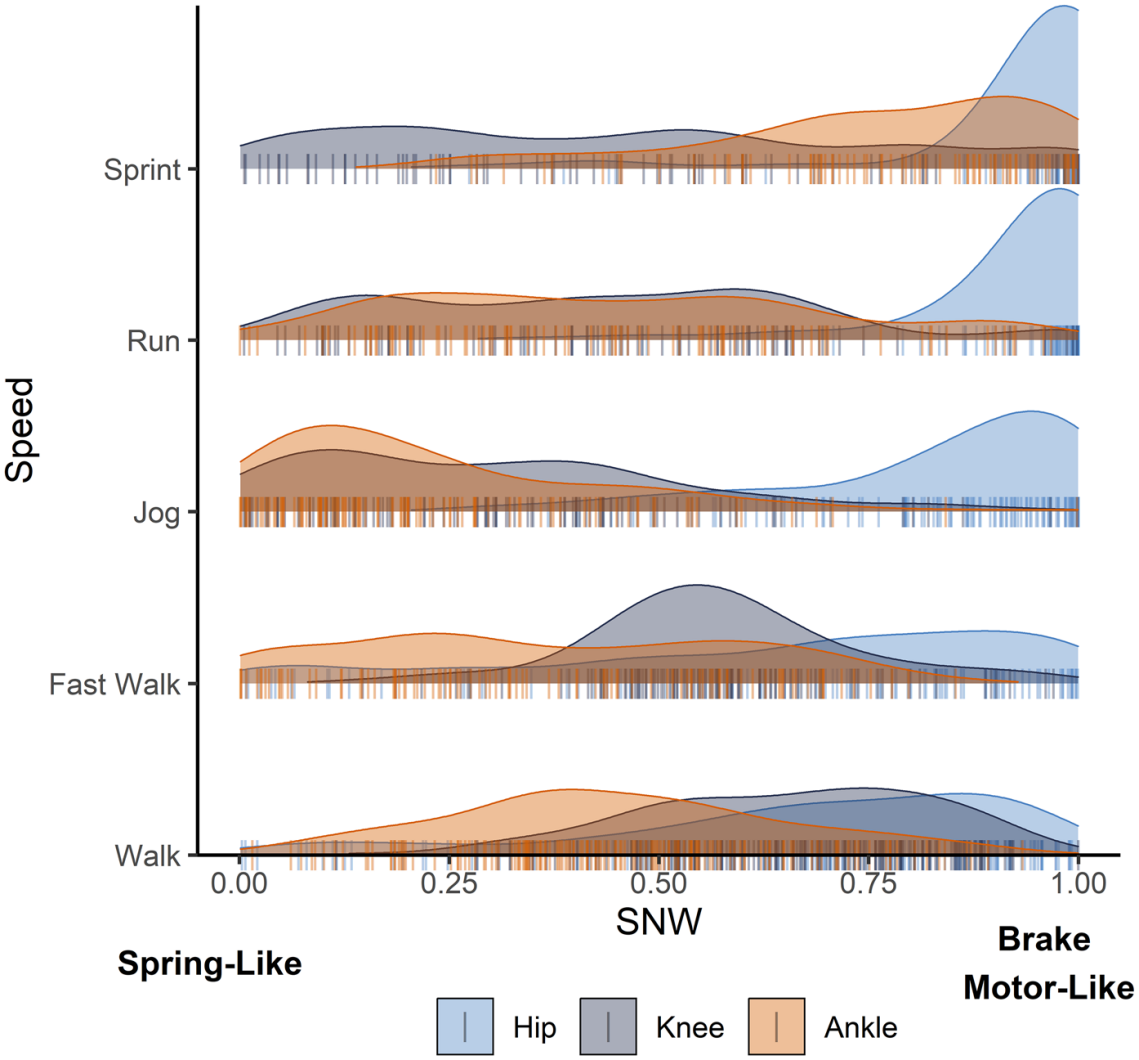
Density plot of individual specific net work (SNW) values for the hip, knee, and ankle for each step, for all subjects, at each speed. Peaks represent the most concentrated distribution of SNW values for each joint, at each speed. Plot rugs (vertical lines) are histograms of SNW values from all subjects and all steps.

**Figure 2:**
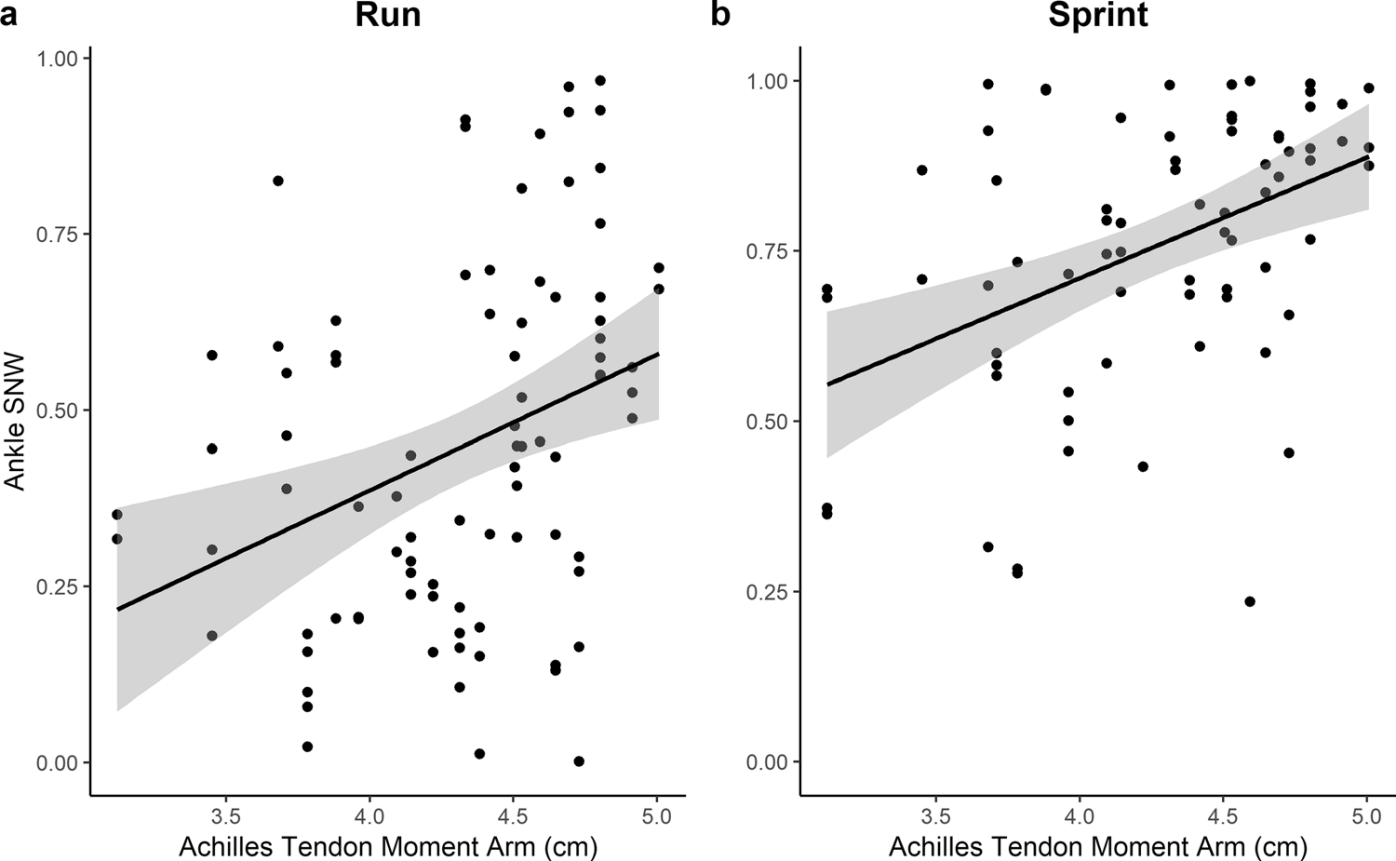
Scatter plots of SNW and the Achilles tendon moment arm length at running (**A**) and sprinting speeds (**B**) against AT moment arm length. The black lines are least squares best fit lines and the gray bands represent 95% confidence intervals.

AT moment arm lengths ranged from 3.12 to 5.01 cm (see boxplot in Supplementary Fig. S1) and are significantly correlated with body mass (r = 0.624, t_[22]_ = 3.748, *p* < 0.001). SNW values at the hip are moderately high in all gaits, whereas values at the knee become more spring-like (i.e., lower values of SNW) in gaits lacking double-limb support periods, such as jogging, running, and sprinting. At the ankle, subjects have the lowest values of SNW when jogging and the highest values when sprinting (see Table 1 for summary statistics for each speed; see Fig. 1 for density plot and histogram of SNW values by joint and speed). We find that AT moment arm length is positively correlated with SNW for running and sprinting gaits, which supports hypothesis H_1_. However, this relationship is not significant for jogging gaits (see Table 2; Fig. 2). Additionally, while sprinting gaits exhibit higher mean SNW values at the ankle compared to walking gaits, the length of the AT moment arm appears to still have a significant effect on spring-like behavior at sprint speeds, but not during walking gaits.

**Table 1:** SNW values for each joint at each speed.

Gait	Froude		Hip SNW		Knee SNW		Ankle SNW	
	Mean	SD	Mean	SD	Mean	SD	Mean	SD
Walk	0.130	0.041	0.669	0.251	0.653	0.170	0.450	0.199
Fast Walk	0.245	0.054	0.640	0.292	0.571	0.153	0.367	0.232
Jog	0.362	0.086	0.834	0.178	0.297	0.220	0.231	0.200
Run	0.662	0.179	0.930	0.124	0.428	0.245	0.447	0.252
Sprint	1.086	0.283	0.935	0.148	0.450	0.299	0.755	0.201

Mean Froude numbers and specific net work (SNW) values with standard deviations (SD) for each joint at each speed.

**Table 2:** Pearson’s correlation coefficient comparisons between ankle SNW, mass-specific stress, and mass-specific elastic energy storage and the Achilles tendon moment arm at different speeds.

Variable	Speed	Statistic	R	*p*-value
**SNW**	Walk	t_144_ = − 0.796	− 0.066	0.786
	Fast walk	t_96_ = − 1.051	− 0.107	0.852
	Jog	t_103_ = 0.675	0.066	0.251
	Run	t_85_ = 3.367	0.343	**0.001**
	Sprint	t_70_ = 4.094	0.440	** < 0.001**
**AT stress/BM**	Run	t_165_ = − 9.185	− 0.582	** < 0.001**
	Sprint	t_102_ = − 5.190	− 0.457	** < 0.001**
**AT tendon energy/BM**	Run	t_165_ = − 4.938	− 0.359	** < 0.001**
	Sprint	t_102_ = − 2.319	− 0.224	**0.011**

Bolded values indicate significance at *p* ≤ 0.05.

Using a mixed-effect multiple regression model with the average moment arm of the GRF vector at the ankle (*R*_*Ankle*_), tendon cross-sectional area (*CSA*), the GRF impulse (*J*), Froude number, and body mass as fixed effect predictors, we find that AT moment arm length does not significantly explain variation in SNW at running speeds. Nevertheless, AT moment arm length and *R*_*Ankle*_ are significant predictors at sprinting speeds (see Table 3). Standardized partial correlation coefficients (i.e., β-weights) demonstrate that *R*_*Ankle*_ plays a more significant role than AT moment arm length, which suggests that tendon elastic energy storage is particularly sensitive to variation in postural variation across steps. However, average *R*_*Ankle*_ is also correlated with AT moment arm length at sprint speeds (r = 0.370, t_[70]_ = 3.336, *p* = 0.001). This result is consistent with the finding that AT moment arm length is correlated with foot length at sprint speeds (i.e., individuals with longer feet have longer AT moment arms; r = 0.684, t_[22]_  = 4.394, *p* < 0.001), which should translate to a longer external moment arm (*R*_*Ankle*_) of the GRF. There were also no sex-based differences when sex was added as a fixed-effect (*p* = 0.680).

**Table 3:** Mixed-effect model of variation in ankle SNW with morphological and kinematic variables as fixed effects at sprint speed.

Predictor variable	β	Statistic	*p*-value
Intercept	− 0.002	F_1,45_ = 0.003	0.954
Ankle moment arm	0.229	F_1,20_ = 15.491	**0.001**
*R*_*Ankle*_	0.336	F_1,45_ = 7.074	**0.011**
Body mass	0.151	F_1,20_ = 0.536	0.473
Froude	0.191	F_1,45_ = 3.271	0.067
*J*	− 0.236	F_1,45_ = 3.520	0.077
*CSA*	0.020	F_1,20_ = 0.001	0.976

Bolded *p*-values indicate significant fixed-effect predictors. β are the partial regression coefficients (i.e., β-weights). *CSA* is the cross-sectional area of the AT. *R*_*Ankle*_ is the mean external moment arm of the ground reaction force. *J* is the GRF impulse.

### Tendon stress and elastic energy storage

**Figure 3:**
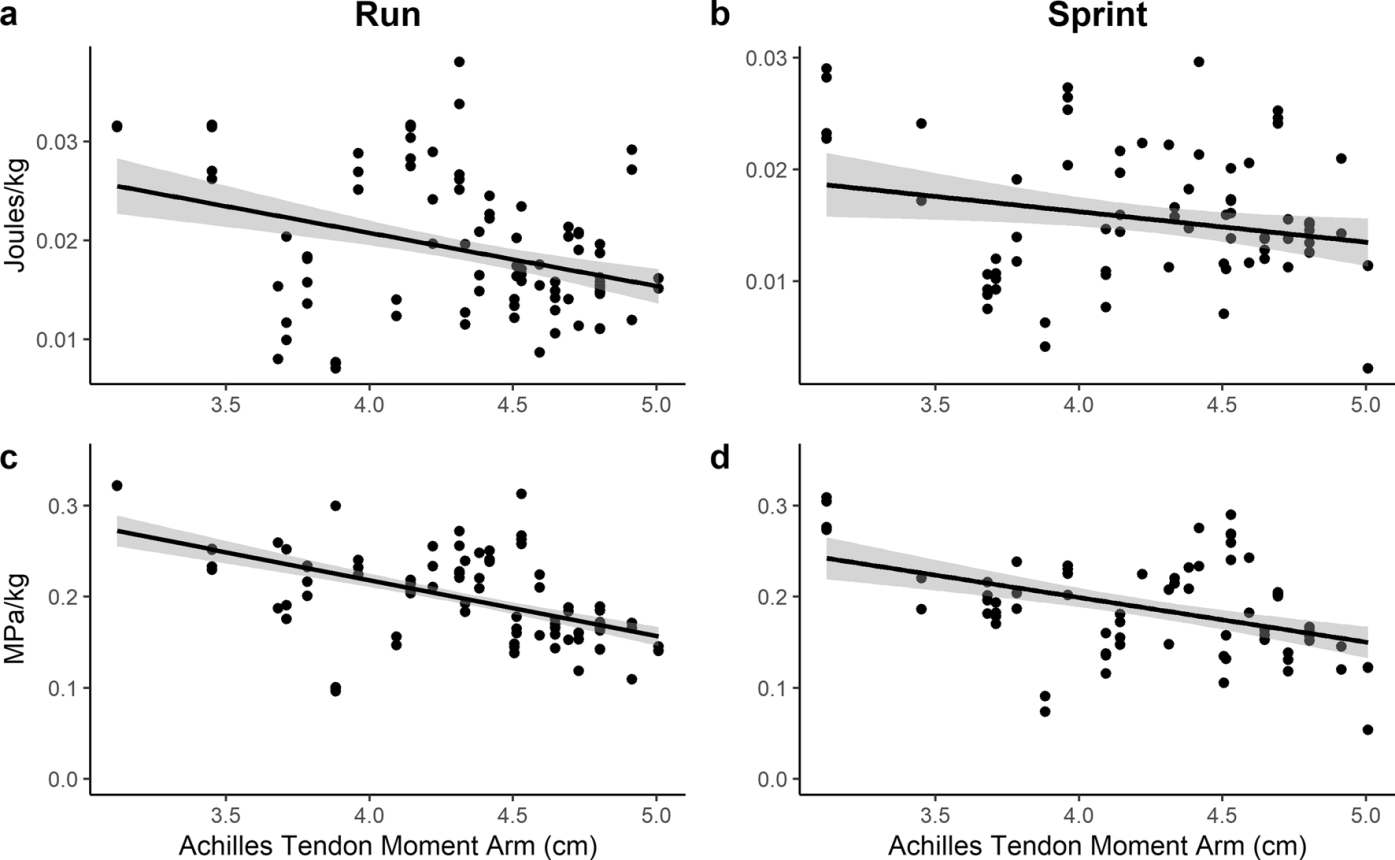
Scatter plots of AT moment arm length and mass-specific tendon stress (MPa/kg) for running (**a**) and sprinting (**b**) trials. Scatter plot of AT moment arm and mass-specific tendon energy storage (Joules/kg) for running (**c**) and sprinting (**d**) trials. The black line is a least squares best fit line and the gray band represents the confidence interval.

Both mass-specific tendon stress (MPa/kg) and mass-specific elastic energy storage (Joules/kg) are negatively correlated with AT moment arm length at running and sprint speeds (see Fig. 3; Table 3). These variables are not correlated at walking, fast walking, and jogging speeds. Subject means are located in Supplementary Tables S1 and S2.

## Discussion

We predicted that subjects with smaller AT moment arm lengths would exhibit lower SNW values in running gaits (i.e., more spring-like joint behavior). The overall pattern is that there is significant variation across gait cycles, but that AT moment arm length does appear to result in greater mass-specific tendon stress, and therefore more spring-like behavior (i.e., lower SNW values), resulting in greater mass-specific energy storage at running and sprinting speeds. The design for this study was derived from a model proposed by Scholz et al.^9^, which predicts that tendon material properties and foot geometry explain inter-individual differences in COL, with AT moment arm length explaining most of this variation. The mixed-effect model analyzing sprint speed kinematics, subject morphology, body size, and tendon CSA, a determinant of tendon strength and stiffness, suggests that CSA does not play a significant role in determining spring-like behavior. Indeed, our results suggest that AT moment arm length and kinematics (i.e., ankle joint posture) play the largest role in determining elastic loading of the tendon. Additionally, the results of the mixed-effect model demonstrate that when holding body size constant, AT moment arm length is still a significant predictor. If subjects had similar tendon properties for their mass, mass-specific measures of elastic loading to the tendon would not vary with AT moment arm length. Therefore, these results provide robust support for the hypothesis that heel allometry is a significant factor in moderating elastic energy storage. It is also worth noting that there is variation about the regression line in ways that differ from scatter about the regression line when looking at COL in previous work^8, 9^. This is likely attributed to step-to-step variation among and between subjects. COL calculations are an average over two minutes, containing multiple steps, whereas values for SNW, tendon stress, and elastic strain energy in this study are averages over stance phase for multiple sequential steps.

This study also builds upon previous work by exploring how speed and AT moment arm length moderates elastic energy storage. AT moment arm length does not explain variation in tendon performance during walking gaits, which is consistent with predictions and results from Scholz et al.^9^ and Raichlen et al.^8^. AT moment arm length does explain variation in tendon performance in running and sprinting gaits, though not in jogging gaits, despite jogging gaits having the lowest mean value for SNW at the ankle for all subjects (0.231). The mean SNW values for running and sprinting were higher, at 0.447 and 0.755, respectively (see Table 1). However, while sprint speed SNW values are more motor/brake-like at sprinting speeds, the relationship between AT moment arm length and SNW is stronger at sprint speeds (r = 0.440, t_[70]_ = 4.094, *p* < 0.001) than running speeds (r = 0.343, t_[85]_ = 3.367, *p* = 0.001) (see Table 2). We interpret these findings to indicate that subjects need to generate more positive work at all lower limb joints as speed increases, but that individuals with short calcanei are nonetheless able to harness relatively more energy from the spring-like return of the AT in running gaits. However, our results demonstrate that AT moment arm length is not correlated with spring-like behavior during walking gaits, which supports previous work showing no correlation between AT moment arm length and metabolic costs in walking gaits^8^.

It is notable that the subject-determined sprint speeds used in this study are on average slower than the treadmill speed used in Scholz et al.^9^ and Raichlen et al.^8^. Subject sprinting speeds in this study are 3.02 ± 0.478 m/s (Froude 1.04 ± 0.309). Subjects from previous studies ran at a sustained pace of 16 km/h (4.44 m/s) on a treadmill, while shod^8, 9^. However, the results from this study suggest a relationship between AT moment arm length and spring-like behavior at speeds above a jog, which may include at 4.44 m/s (16 km/h). The subject population is also different. Raichlen et al.^8^ used trained endurance runners whose 10 K personal best runs were under 36 min and Scholz et al.^9^ used subjects who self-identified as runners. In this study, subjects were recruited based on being recreationally fit but are not necessarily regular runners. Additionally, these previous studies only sampled males, whereas this study has both males and females (and has more females than males) and a larger sample size. Broadly, our comprehensive sampling methods and sample size provide strong empirical support for previous models which suggest that heel morphology moderates AT tendon performance in running gaits.

The mixed-effect model results expand on this relationship by exploring how kinematic variables and tendon *CSA* are correlated with spring-like behavior of the AT. When these variables are included as fixed effects, AT moment arm length is not a significant predictor of SNW at running speeds. However, at sprint speeds both the AT moment arm and the external moment arm of the GRF (*R*_*Ankle*_) are significant predictors, with *R*_*Ankle*_ explaining more variation. Posture and footfall pattern (e.g., mid- vs. forefoot striking) may play a role in elastic loading, perhaps as reflected in the inter- and intra-subject variation in SNW values from step to step. *R*_*Ankle*_ may also play a role in elastic energy storage by altering tendon stiffness depending on foot strike pattern (e.g., heel vs. fore-foot strike). Hof et al.^25^ found that subjects with the highest ankle moments exhibited greater stiffness in the elastic series component of the *m.* *triceps surae*.

Results from tendon stress and estimates of elastic energy storage are consistent with measures of spring-like behavior (i.e., SNW). These results demonstrate that smaller AT moment arm lengths are correlated with higher mass-specific tendon stress values, which in turn result in greater amounts of mass-specific elastic energy storage. These data support previous models and empirical findings demonstrating a correlation between AT moment arm length and COL. The differences between the smallest and largest values of AT moment arm length in our sample are substantial. Values of AT moment arm lengths in our study varied from 3.12 to 5.01 cm, a 37.7% difference that can lead to as much as a 60.7% increase in mass-specific elastic energy storage between subjects with the shortest and longest moment arms.

In this study, tendon stress is calculated using the force impulse (time integrated force). This has the advantage of reflecting force imparted to the AT over the entirety of stance phase. Peak values, by contrast, only reflect instantaneous loads and are more relevant to estimating safety factor and injury risk. Previous work calculated peak stresses of 111 MPa measured from turnbuckles on the AT while running^26^. Stress data measured in this study are similar. The mean value for peak stress from this study (using an inverse dynamics approach) from all subjects is 110.58 MPa at sprint speeds and 101.66 MPa for running speeds (see subject means in Table S1 and S2). There have been a range of estimates for failure stress in the AT^26–30^. Wren et al.^30^ found a mean failure stress of 79 MPa when straining Achilles tendons at 1–10% per second. While the mean stress values from this study exceed the mean failure stress from Wren et al.^30^, loading rates measured in this study were not of the same magnitude and duration as tests for plastic deformation and failure. Overall, the AT has similar material properties to other tendons, but receives a much higher load during running, with an estimated safety factor of 1.5, compared to other tendons that have safety factors of ~ 4^28, 31^. However, because of the relationship with AT moment arm length and tendon stress, it is possible that foot geometry may be predictive of risk for tendinopathy. Achilles tendinopathy (i.e., pain and swelling of the Achilles tendon) is one of the most common sports-related injuries^32^. Ex vivo data from human ATs suggest that excessive tendon strain (the result of tendon stress) is a primary factor responsible for tendon damage, and that repetitive loading contributes to AT injury and tendinopathy^33^. Moreover, a majority of AT ruptures are sub-clinical^34^, which suggests that intrinsic factors may play a significant role in explaining tendon strain and predicting risk for injury. Future work should explore this association. Additionally, athletic training is linked with changes to AT material properties which is relevant to injury prevention and may play a role in the capacity for elastic energy storage in the AT^29, 35^.

Previous work has characterized elastic energy storage of the *m.* *gastrocnemius * and Achilles tendon during walking and running gaits using inverse dynamics and ultrasonography^13, 14, 16–19, 36, 37^. This is the first study to measure how AT moment arm length moderates tendon stress and elastic energy storage. However, it is important to note there are some limitations imposed by the study design and that a detailed understanding of the relationship between foot geometry and the spring-like function of the AT requires further work. In particular, future work should incorporate non-invasive imaging methods (e.g., ultrasonography) to measure instantaneous changes in AT length and moment arm dimensions. Increased temporal precision may offer greater insight into how foot geometry may impact elastic energy storage at different points in stance phase. Indeed, previous work has demonstrated that accounting for tendon curvature at different points of stance phase may provide more precision in both measurements of tendon dimensions and moment arm length^38^. Rasske et al.^39^ found that during walking gaits, AT moment arm length changed 10% at toe-off relative to heel strike. Recent work by Harkness-Armstrong et al. found that assuming a straight (as opposed to curved) AT led to larger estimates of moment arm length than actual size^38^. In this study, joint work was calculated using moment arm lengths which vary with joint angle (and scaled to each subject; see “Methods” section). However, future work which measures how the AT moment arm changes with joint angle, load, and speed for each individual using ultrasonography may increase precision for estimating joint work across stance phase^40–42^. For statistical comparisons of spring-like behavior (SNW), joint stress, strain, and elastic energy storage, a static measure of AT moment arm length was used following Scholz et al.^9^. Previous work from other studies suggests that this method provides reliable measures of the AT moment arm^43^. However, exploring how tendon performance changes at different points of stance phase in relation to ankle moment arm length may also provide further insight into this relationship.

Exploring how AT moment arm length moderates tradeoffs between muscle work and elastic energy storage would also provide further clarification. For example, holding all else equal, a shorter moment arm should result in less muscle fiber work (shortening) for a given joint rotation. Any decrease in muscle fiber work should result in a reduction in metabolic cost, which is consistent with results from previous work demonstrating a negative correlation between AT moment arm length and COL^8, 9^. The model proposed by Scholz et al.^9^ (and explored in this study), also predicts that shorter AT moment arms will result in increased tendon load. However, any increase in tendon load should be a result of an increase in muscle force. This creates a potential conflict in interpreting the relationship between AT moment arm length, metabolic cost, and muscle force. The results from this study suggest that shorter AT moment arms increase tendon load and elastic energy storage such that the balance of this tradeoff still favors a shorter AT moment arm, holding all else equal. However, future work which explores how AT moment arm length moderates muscle fiber work, muscle force, tendon load, elastic energy storage, and metabolic cost would provide further insight into the tradeoffs imposed between muscle force and elastic energy storage in individuals with shorter AT moment arm lengths.

Individual measures of tendon material properties and elastic modulus may also offer greater clarification on how anatomy predicts elastic energy storage. Previous research indicates that material properties of the AT and force generating capacity of muscles varies between individuals and is correlated with elastic energy storage^15, 18, 44–46^. Additionally, tendon CSA may change with deformation of the tendon as force is applied throughout stance phase and should be accounted for in future studies^47^. The stiffness of the *m.* *gastrocnemius* aponeurosis is also a significant factor in contributing to muscle work and elastic energy storage^17, 48–50^. Methods which measure gearing and muscle–tendon stiffness, which vary with speed and torque development, also have been shown to influence elastic energy storage at different points of stance phase, and therefore may also be moderated by foot geometry like the AT moment arm^13, 14, 19, 36^.

In conclusion, the results from this study suggest that there is a significant correlation between moment arm length and spring-like behavior of the AT. This spring-like behavior also corresponds with greater tendon stress and elastic energy storage in subjects with smaller AT moment arm sizes. Overall, our findings provide empirical mechanical support for the energetic model proposed by Scholz et al.^9^, suggesting that calcaneal length may be an important skeletal determinant of variation in COL during human bipedal running.

## Methods

To test how AT moment arm length predicts spring-like behavior of the AT, we collected morphometric, kinematic, kinetic, and ultrasound data from 24 recreationally fit adults (see Table 4 for summary information). Subjects were asked to walk, fast walk, jog, run, and sprint at self-determined speeds across 6 force platforms embedded in the floor (2 platforms in width, 3 platforms in length), which allowed for multiple support phases to be measured per trial. Subjects were unshod and repeated each speed three times. Subject speeds were calculated as the mean velocity of the marker placed on the greater trochanter of each leg during the support phase of each gait cycle, which were then standardized using Froude numbers (see Eq. (1))^51^:

**Table 4:** Subject morphometrics and summary statistics.

Sex		Age	Body mass	AT moment arm	AT cross-sectional area
Male	Female	Years	kg	cm	cm^2^
7	17	23.50 ± 2.80	64.15 ± 9.84	4.27 ± 0.48	0.61 ± 0.15

Subject morphometrics and statistics presented as the number of males and females and all numeric variables represent the mean and the standard deviation (AT: Achilles tendon).

1 $$\frac{{velocity}^{2}}{gravitational\, acceleration \cdot hip \,height}$$

### Ultrasound

**Figure 4:**
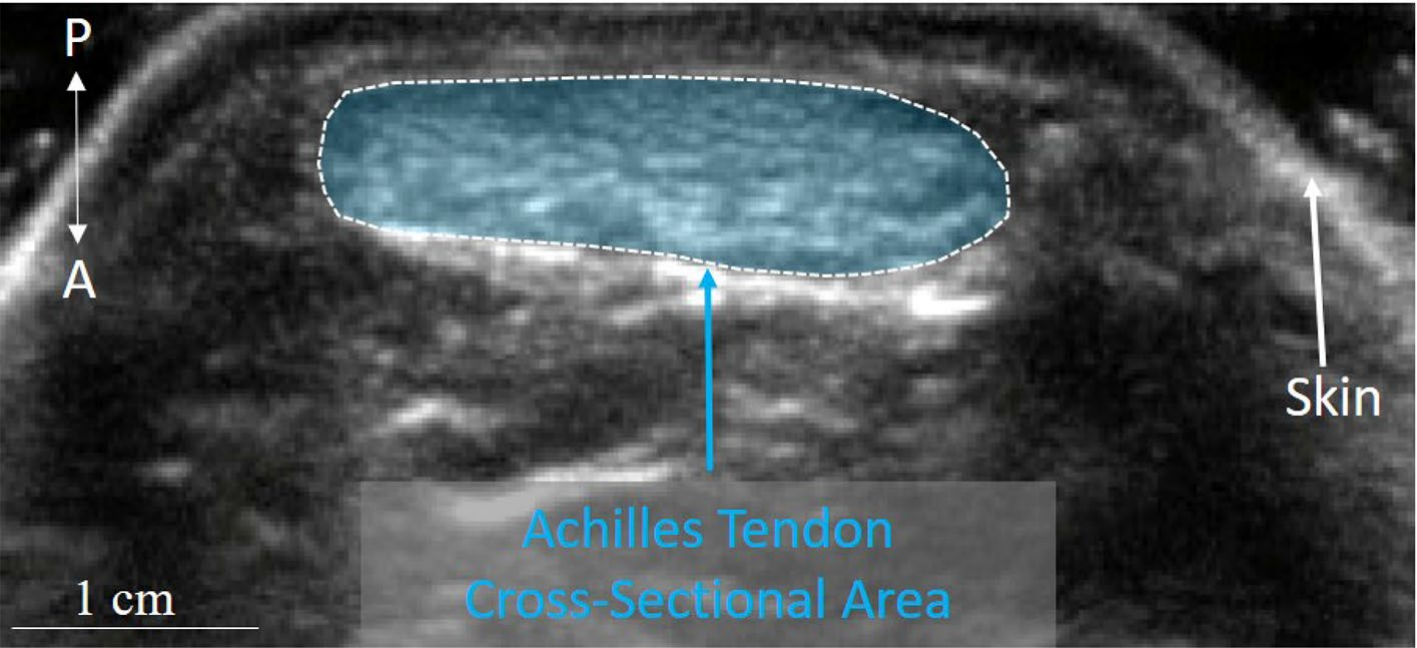
The cross-sectional area of the Achilles tendon measured using ultrasound. Example of the cross-sectional area of the Achilles tendon measured at the level of the malleoli using B-mode ultrasound. The area of the tendon outlined by the dashed white line and shaded in blue was calculated using ImageJ. A is anterior and P is posterior. The hyperechoic region anterior to the shaded ellipse around the tendon is Kager’s fat pad.

The cross-sectional area of the AT was measured with B-mode ultrasound (SonoSite M-Turbo, Fujifilm SonoSite, Bothell, Washington, USA) using a 6 cm linear array transducer operating at 15 MHz (HFL50; Fujifilm SonoSite, Bothell, Washington, USA), with a depth set to 2.7 cm. The resolution of the ultrasound image is 100 pixels/cm. To measure the cross-sectional area of the AT, the probe was placed in a transverse plane at the level of the malleoli while subjects were prone on an examination table and the ankle was fixed at 160° (plantarflexed), which was measured with a goniometer. The cross-sectional area of the tendon was measured in ImageJ, by tracing the outline of the tendon using the polygon area selection tool and measuring the area^52^ (see Fig. 4). To measure tendon length, subjects lay prone with their foot in a neutral position (i.e., 90°). Using the same ultrasonographic technique, the myotendinous junction of the medial head of *m.* *gastrocnemius* on the left and right leg was located by placing it in the center of the image*.* A washable marker was used to place a dot on the skin at this location using the midpoint guide on the probe. This same procedure was used to determine the most inferior extent of the insertion of the tendon on the calcaneal tuberosity. Tendon length was measured using a flexible measuring tape and is defined as the distance of the myotendinous junction to the insertion on the calcaneal tuberosity. Tendon length for each subject is an average of the lengths of the left and right sides.

### Morphometrics

Subject body mass and height were recorded using a digital scale and segment lengths (hip height and foot length) were measured using a flexible measuring tape. Hip height was defined as the distance from the greater trochanter to the floor while standing. Foot length was defined as the most anterior point of the first digit to the most posterior point of the calcaneal tuberosity. Subject values for segment lengths are an average of the left and right sides.

To compare tendon performance data collected in this study to previous work, we measured the moment arm of the AT using photographic methods^9^. Subjects stood with their ankle at an angle of 90° (where the leg is perpendicular to the foot) on a board that was affixed with a measuring tape (see Supplementary Fig. S2). The location of the footboard and camera were standardized and the malleoli were centered in the image to reduce the effects of parallax distortion. The moment arm was defined as the most medially or laterally prominent point of the medial or lateral malleolus, respectively, to the most posterior edge of the skin covering the tendon. Distances were measured in ImageJ^52^ using the measuring tape as a scale. The medial and lateral values for both the left and right foot were averaged from three repeated measurements, then those averages from the medial and lateral side were averaged for each subject to obtain a single mean value for the AT moment arm. These moment arm values are used as the dependent variable when exploring the relationship between kinematic variables and elastic loading. See Table 4 for subject summary statistics and Supplementary Fig. S1 for a boxplot of subject AT moment arm lengths.

### Kinematics and kinetics

Subjects were fitted with retro-reflective markers placed on joint centers of the hip, knee, and ankle, in addition to other standardized locations (see Supplementary Fig. S3). Subjects were asked to walk, fast walk, jog, run, and sprint at self-determined speeds. Runway distances were sufficient for subjects to reach steady state locomotion when crossing force plates. Force data were collected using six force platforms (BTS P6000D, BTS Bioengineering Corp., Quincy, MA, USA) that were embedded in the floor, with a sampling rate of 1 kHz. Kinematic data were recorded using a 12-camera motion capture system (BTS Bioengineering Corp., Quincy, MA, USA), with a sampling rate of 500 Hz. Force data were deprecated to 500 Hz to synchronize force and kinematic data which were processed using custom routines in MATLAB (version 2018b; Mathworks, Natick, MA, USA). A zero-lag Butterworth, low-pass filter was used to smooth kinematic data (fourth-order, with a 6 Hz cutoff) and force data (fourth-order, with a 100 Hz cutoff).

An inverse dynamics approach was used to calculate joint work for the hip, knee, and ankle over the support phase of each gait cycle using 2D kinematics and ground reaction forces (GRF). We calculated the external moment arm of the GRF (R) as the fore-aft and vertical distance from the center of pressure (COP). Net joint moments were calculated using the GRF vector, limb segment accelerations, and joint moments following Biewener et al.^53^ and Winter^54^. Because mediolateral forces have a negligible impact on joint moments, these moments were ignored in the calculation of biomechanical variables. Net joint moments, *M,* were determined for each kinematic frame, at the hip, knee, and ankle joint using the free-body method described in Winter^54^. Limb segment accelerations were calculated using the second-order finite differences method^54^. Extensor muscle forces (*F*_*ankle*_, *F*_*knee*_, and *F*_*hip*_) to generate these moments were determined by solving a system of equations from Biewener et al.^53^:

2 $${\text{M}}_{{{\text{ankle}}}} \, = \,{\text{F}}_{{{\text{ankle}}}} \cdot{\text{r}}_{{{\text{Ankle}}}}$$

3 $${\text{M}}_{{{\text{Knee}}}} \, = \,{\text{F}}_{{{\text{Knee}}}} \cdot {\text{r}}_{{{\text{Knee}}}} {-}\,{\text{F}}_{{{\text{G}},{\text{Knee}}}} \cdot {\text{r}}_{{{\text{G}},{\text{Knee}}}} {-}{\text{F}}_{{{\text{H}},{\text{Knee}}}} \cdot {\text{r}}_{{{\text{H}},{\text{Knee}}}}$$

4 $${\text{M}}_{{{\text{Hip}}}} = {\text{ F}}_{{{\text{Hip}}}} \cdot {\text{r}}_{{{\text{Hip}}}} \,{-}\,{\text{F}}_{{{\text{RF}},{\text{Hip}}}} \cdot {\text{r}}_{{{\text{RF}},{\text{Hip}}}}$$

Subtracted terms represent flexor actions of bi-articular muscles gastrocnemius (G), hamstrings (H), and rectus femoris (RF). Flexor and extensor moments were calculated assuming the force produced by each muscle is proportional to its physiological cross-sectional area (PCSA)^53^. Muscle force impulse at the hip, knee, and ankle was calculated as the finite integral of instantaneous muscle force throughout each support phase. Human extensor moment arms, *r*, for the hip, knee, and ankle, were calculated as instantaneous values that vary with joint angle using equations from the literature for the hip and knee from Visser^55^ and ankle from Rugg et al.^56^.

To calculate the average moment arm of the GRF vector (R) at the ankle (*R*_*Ankle*_) for each support phase, we used the GRF impulse (*J*), AT moment arm length (*r*_*Ankle*_; calculated following Rugg et al.^56^) and the ankle force impulse (*F*_*Impulse*_) following equations from Biewener et al.^53^ [see Eq. (5)]:

5 $${R}_{Ankle} =\frac{{\int }_{{t}_{1}}^{{t}_{2}}GRF \cdot {r}_{Ankle}}{{\int }_{{t}_{1}}^{{t}_{2}}{F}_{Ankle}}$$

Using equations from Winter^54^, we calculated work, which is the finite integral of joint power (Watts/kg) over time, at the hip, knee, and ankle joint. Positive and negative values for work were used to calculate SNW, which is a ratio of the sum of positive and negative work to the sum of the total work, to characterize the spring-like behavior of lower limb joints [see Eq. (6)]*.* Negative values (instantaneous values less than 0) for work are summed as *Work*_*Neg*_ and positive values for work are summed as *Work*_*Pos*_.

6 $$\frac{|{Work}_{Pos}+ {Work}_{Neg}|}{|{Work}_{Pos}|+|{Work}_{Neg}|}$$

### Tendon stress, strain, and energy storage

We calculate tendon stress, which is defined as the force impulse imparted to the AT, scaled to the cross-sectional area of the AT, for each subject:

7 $${Tendon}_{stress} =\frac{{\int }_{{t}_{1}}^{{t}_{2}}{F}_{Ankle}}{{Tendon}_{CSA}}$$

Here, *F*_*Ankle*_ is the instantaneous value of muscle force at the ankle joint for each support phase of a gait cycle (ankle force impulse, *F*_*Impulse*_), and t_1_ and t_2_ represent the beginning and end of the support phase interval. Tendon *CSA* is calculated as the cross-sectional area and converted to square meters (m^2^) from ultrasound images of the AT for each subject (see ultrasound methods).

Tendon strain is calculated by dividing tendon stress by the elastic modulus of the AT. Here, we use 819 MPa for the elastic modulus, which is the mean value of a sample of human ATs from Wren et al.^30^. Tendon strain is then multiplied by the resting tendon length for each subject (see ultrasound methods) to calculate the estimated change in the length of the tendon. Strain energy, or the amount of elastic energy storage in the tendon, is modeled following Hooke’s law [see Eq. (8)].

8 $$W =\frac{1}{2}{F}_{Ank}\Delta L$$

Here, *F*_*Ankle*_ is the ankle force impulse and *L* is the change in length of the AT. Following Moore et al.^23^, we multiplied the amount of energy recovered by 0.93, which is an estimate of tendon resilience (i.e., 93%).

### Statistics

All statistical analyses for this study were conducted in the R statistical platform (version 3.6.1, 'Action of the Toes')^57^. We used one-tailed Pearson product-moment correlations to test for positive association between SNW and AT moment arm lengths and negative association between AT moment arm lengths and stress and elastic energy storage. Mixed-effect models were used to explore the correlation between SNW and AT moment arm lengths with kinematic and morphometric variables as fixed effects (using the lme function)^58^. A mixed-effect model is used for testing hypotheses in this study as it allows for adjustment of degrees of freedom to account for variation between individuals, and error terms to account for repeated measures of multiple steps from the same subject. Raw variates were scaled and centered (converted to z-scores) to permit direct comparisons of resulting partial regression coefficients (β weights) to allow for comparison of which predictors best explain variance in the dependent variable. Results for all tests were significant at *p* < 0.05. Plots were made using ggplot2^59^.

### Institutional oversight and compliance

Institutional Review Board approval was obtained by Campbell University (Protocols 376 and 472) and all study methods and procedures in this study followed the approved protocols and all IRB guidelines. Informed consent was obtained prior to subject participation.

## Supplementary Information

Supplementary Information.

## Data Availability

Data and the R code used for statistical analysis and generating figures and tables in this study are available at: https://github.com/adfoster/achillestendon
