# Supplementary material for: Shorter heels are linked with greater elastic energy storage in the Achilles tendon
Source: Sci Rep. 2021 Apr 30;11:9360. doi: 10.1038/s41598-021-88774-8 (PMC8087768; doi:10.1038/s41598-021-88774-8)
Supplement: Supplementary file 1 — Supplementary Information. [file 41598_2021_88774_MOESM1_ESM.docx]

**Supplemental Information**

Shorter heels are linked with greater elastic energy storage in the Achilles tendon

Foster AD, Block B, Capobianco III F, Peabody JT, Puleo NA, Vegas A, Young JW.

**Supplementary Figure 1. Boxplot of Achilles Tendon moment arm lengths**


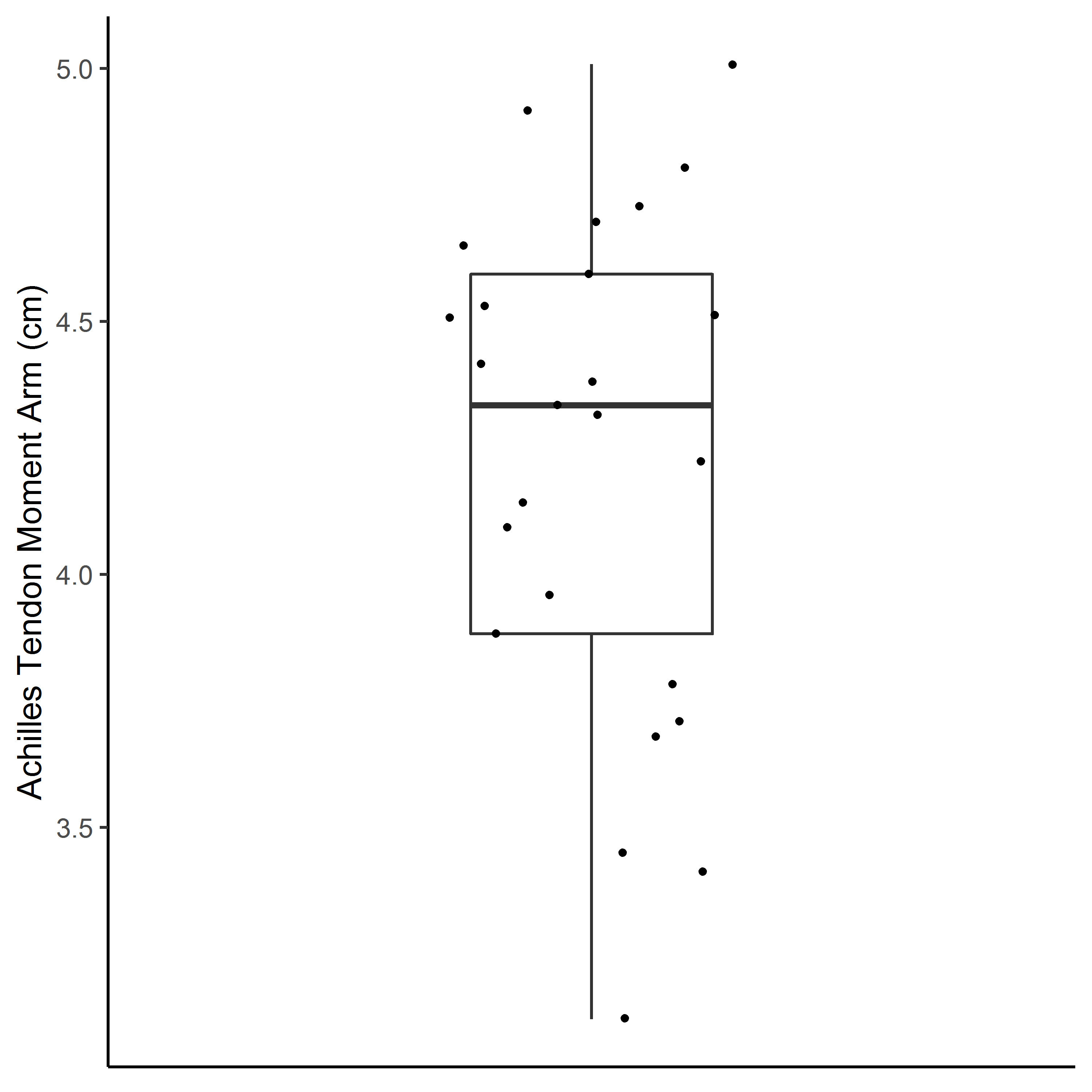


Combined boxplot and dotplot to show distribution of AT moment arm lengths among subjects. The horizontal line indicates the median and the outer boundaries of the box represent the interquartile range. The dots represent individual subject values for AT moment arm length.

**Supplementary Figure 2. Measurement of the Achilles tendon moment arm**

**
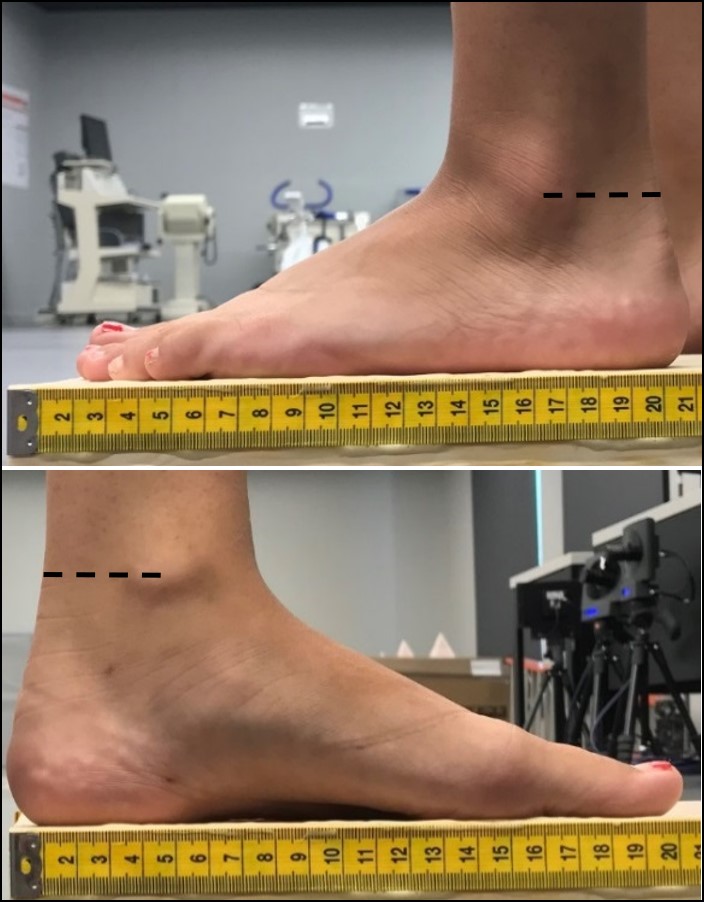
**

Photographic methods were used to measure the Achilles tendon moment arm following Scholz et al.^1^. The dashed lines represents the distance between the most medial or lateral point on the medial or lateral malleolus to the most posterior point on the Achilles tendon. The moment arm length for each subject is a mean of lateral and medial lengths from the left and right foot (see Methods).

**Supplementary Figure S3 – Kinematic marker set**

**
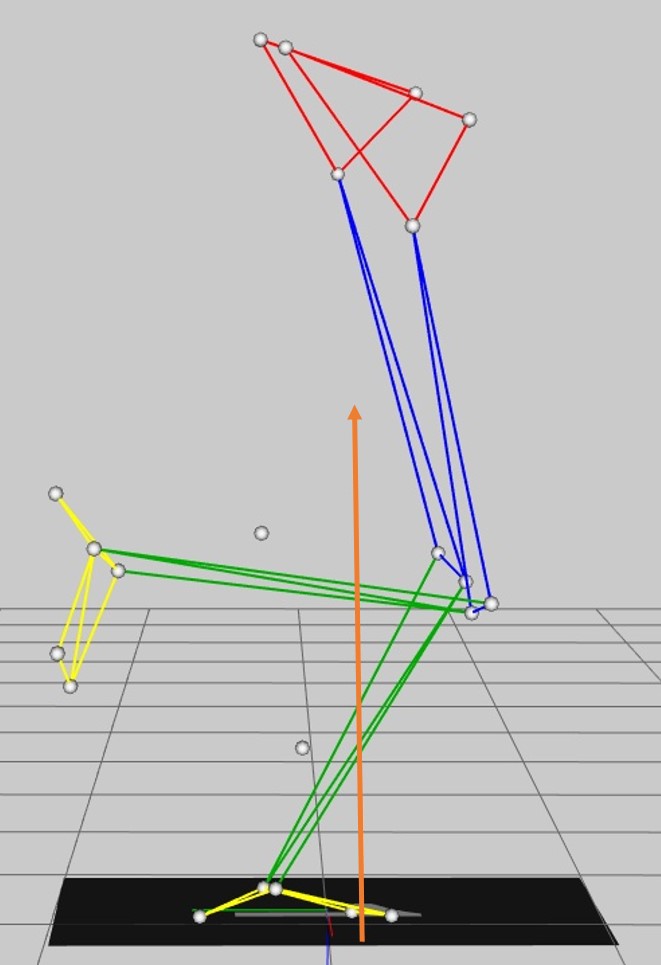
**

Markers were place on joint centers (hip, knee, and ankle), as well as the anterior superior iliac spine (ASIS), posterior superior iliac spine (PSIS), the most posterior point on the calcaneal tuberosity, and the heads of the 1^st^ and 5^th^ metatarsals. The markers not joined by segments are the location of the myotendinous junction of the medial *gastrocnemius m.* and the Achilles tendon. This image was created in Mokka (version 0.6.2; https://biomechanical-toolkit.github.io/mokka/).

**Supplemental Tables**

**Supplementary Table S1. Mean tendon stress and elastic energy storage by subject and speed**

|  |  | **Tendon Stress (Mpa)** | | **Tendon Energy (Joules)** | | **Tendon Energy (Joules/kg)** | |
| --- | --- | --- | --- | --- | --- | --- | --- |
| *Subject* | *Gait* | *Mean* | *SD* | *Mean* | *SD* | *Mean* | *SD* |
| Subject1 | Sprint | 11.89 | 2.84 | 1.12 | 0.52 | 0.0167 | 0.0078 |
| Subject1 | Run | 16.17 | 1.46 | 2.03 | 0.37 | 0.0303 | 0.0055 |
| Subject2 | Sprint | 5.93 | 0.88 | 0.38 | 0.11 | 0.0052 | 0.0015 |
| Subject2 | Run | 7.15 | 0.15 | 0.54 | 0.02 | 0.0075 | 0.0003 |
| Subject3 | Sprint | 12.42 | 1.54 | 0.89 | 0.22 | 0.0151 | 0.0037 |
| Subject3 | Run | 13.28 | 0.73 | 1.01 | 0.11 | 0.0171 | 0.0018 |
| Subject4 | Sprint | 8.05 | 0.40 | 0.46 | 0.05 | 0.0103 | 0.0010 |
| Subject4 | Run | 9.56 | 1.66 | 0.67 | 0.23 | 0.0148 | 0.0051 |
| Subject5 | Sprint | 11.72 | 0.88 | 0.88 | 0.13 | 0.0165 | 0.0025 |
| Subject5 | Run | 12.08 | 1.05 | 0.94 | 0.16 | 0.0176 | 0.0031 |
| Subject6 | Sprint | 7.17 | 2.85 | 0.58 | 0.38 | 0.0077 | 0.0050 |
| Subject6 | Run | 10.83 | 0.21 | 1.18 | 0.05 | 0.0156 | 0.0006 |
| Subject7 | Sprint | 7.68 | 1.32 | 0.60 | 0.20 | 0.0093 | 0.0032 |
| Subject7 | Run | 9.14 | 0.30 | 0.84 | 0.05 | 0.0130 | 0.0008 |
| Subject8 | Sprint | 8.23 | 1.02 | 0.64 | 0.16 | 0.0104 | 0.0026 |
| Subject8 | Run | 9.32 | 0.33 | 0.81 | 0.06 | 0.0132 | 0.0009 |
| Subject9 | Sprint | 13.21 | NA | 1.32 | NA | 0.0224 | NA |
| Subject9 | Run | 13.71 | 1.32 | 1.43 | 0.27 | 0.0243 | 0.0046 |
| Subject10 | Sprint | 10.28 | 1.40 | 1.37 | 0.37 | 0.0176 | 0.0047 |
| Subject10 | Run | 11.27 | 2.55 | 1.70 | 0.71 | 0.0218 | 0.0091 |
| Subject11 | Sprint | 9.70 | 1.24 | 0.91 | 0.23 | 0.0135 | 0.0034 |
| Subject11 | Run | 11.34 | 0.56 | 1.23 | 0.12 | 0.0183 | 0.0018 |
| Subject12 | Sprint | 14.73 | 0.90 | 1.31 | 0.16 | 0.0259 | 0.0031 |
| Subject12 | Run | 16.26 | 0.02 | 1.59 | 0.00 | 0.0315 | 0.0001 |
| Subject13 | Sprint | 13.72 | 0.25 | 1.02 | 0.04 | 0.0162 | 0.0006 |
| Subject13 | Run | 13.10 | 1.85 | 0.95 | 0.27 | 0.0150 | 0.0042 |
| Subject14 | Sprint | 11.25 | 1.34 | 1.14 | 0.27 | 0.0206 | 0.0049 |
| Subject14 | Run | 13.39 | 0.62 | 1.61 | 0.15 | 0.0291 | 0.0027 |
| Subject15 | Sprint | 12.01 | 2.40 | 0.91 | 0.36 | 0.0161 | 0.0063 |
| Subject15 | Run | 10.84 | 1.80 | 0.74 | 0.24 | 0.0131 | 0.0042 |
| Subject16 | Sprint | 10.53 | 0.70 | 0.48 | 0.06 | 0.0090 | 0.0012 |
| Subject16 | Run | 11.84 | 2.22 | 0.62 | 0.23 | 0.0116 | 0.0042 |
| Subject17 | Sprint | 13.51 | 0.81 | 1.51 | 0.17 | 0.0249 | 0.0029 |
| Subject17 | Run | 14.17 | 0.40 | 1.66 | 0.09 | 0.0273 | 0.0015 |
| Subject18 | Sprint | 9.92 | 0.80 | 1.04 | 0.17 | 0.0135 | 0.0022 |
| Subject18 | Run | 11.07 | 1.53 | 1.31 | 0.34 | 0.0170 | 0.0045 |
| Subject19 | Sprint | 13.48 | 1.19 | 1.46 | 0.26 | 0.0176 | 0.0031 |
| Subject19 | Run | 17.67 | 0.49 | 2.50 | 0.14 | 0.0301 | 0.0017 |
| Subject20 | Sprint | 15.72 | 0.19 | 1.92 | 0.05 | 0.0246 | 0.0006 |
| Subject20 | Run | 13.59 | 1.51 | 1.44 | 0.31 | 0.0186 | 0.0040 |
| Subject21 | Sprint | 10.56 | 0.44 | 0.92 | 0.08 | 0.0139 | 0.0012 |
| Subject21 | Run | 11.01 | 1.03 | 1.01 | 0.18 | 0.0152 | 0.0028 |
| Subject22 | Sprint | 17.91 | 1.21 | 1.14 | 0.15 | 0.0168 | 0.0023 |
| Subject22 | Run | 18.94 | 1.73 | 1.28 | 0.24 | 0.0189 | 0.0035 |
| Subject23 | Sprint | 14.18 | 1.65 | 1.42 | 0.33 | 0.0255 | 0.0059 |
| Subject23 | Run | 13.55 | 0.36 | 1.29 | 0.07 | 0.0231 | 0.0012 |
| Subject24 | Sprint | 11.12 | 0.33 | 0.92 | 0.05 | 0.0132 | 0.0008 |
| Subject24 | Run | 11.46 | 0.70 | 0.98 | 0.12 | 0.0140 | 0.0017 |

Summary table of subject means and standard deviations (SD) at sprint and running speeds for tendon stress, mass-specific tendon stress, elastic energy storage, and mass-specific elastic energy storage. All tendon stress and elastic energy storage values are calculated using the ankle force impulse. An ‘NA’ for the SD means that only one step with useable data was recorded for that speed for that subject.

**Supplementary Table S2. Peak tendon stress and elastic energy storage by subject and speed**

|  |  | **Tendon Stress (Mpa)** | | **Tendon Stress (Mpa/kg)** | | **Tendon Energy (Joules)** | | **Tendon Energy (Joules/kg)** | |
| --- | --- | --- | --- | --- | --- | --- | --- | --- | --- |
| *Subject* | *Gait* | *Mean* | *SD* | *Mean* | *SD* | *Mean* | *SD* | *Mean* | *SD* |
| Subject1 | Sprint | 235.56 | 128.56 | 3.52 | 1.92 | 491.18 | 466.65 | 7.331 | 6.965 |
| Subject1 | Run | 139.64 | 24.31 | 2.08 | 0.36 | 153.89 | 51.68 | 2.297 | 0.771 |
| Subject2 | Sprint | 63.25 | 7.02 | 0.87 | 0.10 | 42.52 | 9.38 | 0.587 | 0.130 |
| Subject2 | Run | 52.84 | 1.30 | 0.73 | 0.02 | 29.51 | 1.45 | 0.408 | 0.020 |
| Subject3 | Sprint | 102.33 | 9.33 | 1.73 | 0.16 | 60.10 | 11.02 | 1.019 | 0.187 |
| Subject3 | Run | 84.48 | 6.63 | 1.43 | 0.11 | 40.93 | 6.37 | 0.694 | 0.108 |
| Subject4 | Sprint | 71.63 | 10.53 | 1.59 | 0.23 | 37.16 | 11.26 | 0.824 | 0.250 |
| Subject4 | Run | 67.85 | 12.11 | 1.50 | 0.27 | 33.51 | 12.32 | 0.743 | 0.273 |
| Subject5 | Sprint | 110.59 | 0.58 | 2.07 | 0.01 | 77.98 | 0.81 | 1.463 | 0.015 |
| Subject5 | Run | 109.94 | 14.61 | 2.06 | 0.27 | 77.97 | 19.72 | 1.463 | 0.370 |
| Subject6 | Sprint | 74.24 | 19.96 | 0.98 | 0.26 | 58.30 | 27.59 | 0.768 | 0.364 |
| Subject6 | Run | 84.22 | 4.54 | 1.11 | 0.06 | 71.68 | 7.71 | 0.944 | 0.102 |
| Subject7 | Sprint | 68.13 | 15.25 | 1.06 | 0.24 | 47.47 | 20.73 | 0.739 | 0.323 |
| Subject7 | Run | 75.56 | 4.12 | 1.18 | 0.06 | 57.07 | 6.30 | 0.889 | 0.098 |
| Subject8 | Sprint | 74.31 | 7.97 | 1.20 | 0.13 | 52.01 | 11.41 | 0.843 | 0.185 |
| Subject8 | Run | 79.72 | 4.44 | 1.29 | 0.07 | 59.44 | 6.61 | 0.963 | 0.107 |
| Subject9 | Sprint | 98.52 | NA | 1.67 | NA | 73.27 | NA | 1.244 | NA |
| Subject9 | Run | 99.90 | 7.98 | 1.70 | 0.14 | 75.65 | 11.89 | 1.285 | 0.202 |
| Subject10 | Sprint | 140.46 | 22.56 | 1.81 | 0.29 | 256.52 | 81.35 | 3.302 | 1.047 |
| Subject10 | Run | 105.35 | 37.29 | 1.36 | 0.48 | 154.37 | 92.66 | 1.987 | 1.193 |
| Subject11 | Sprint | 85.39 | 20.07 | 1.27 | 0.30 | 71.61 | 32.76 | 1.066 | 0.488 |
| Subject11 | Run | 83.61 | 3.90 | 1.24 | 0.06 | 66.90 | 6.28 | 0.996 | 0.093 |
| Subject12 | Sprint | 101.71 | 9.25 | 2.01 | 0.18 | 62.70 | 11.25 | 1.239 | 0.222 |
| Subject12 | Run | 104.18 | 3.57 | 2.06 | 0.07 | 65.41 | 4.48 | 1.293 | 0.089 |
| Subject13 | Sprint | 138.28 | 3.33 | 2.19 | 0.05 | 103.75 | 4.99 | 1.642 | 0.079 |
| Subject13 | Run | 128.17 | 21.03 | 2.03 | 0.33 | 90.70 | 29.23 | 1.435 | 0.463 |
| Subject14 | Sprint | 98.17 | 16.30 | 1.77 | 0.29 | 87.68 | 28.72 | 1.580 | 0.517 |
| Subject14 | Run | 91.92 | 9.25 | 1.66 | 0.17 | 76.41 | 15.98 | 1.377 | 0.288 |
| Subject15 | Sprint | 119.67 | 15.05 | 2.11 | 0.27 | 89.34 | 22.30 | 1.579 | 0.394 |
| Subject15 | Run | 102.09 | 13.32 | 1.80 | 0.24 | 65.25 | 16.50 | 1.153 | 0.292 |
| Subject16 | Sprint | 124.04 | 20.73 | 2.34 | 0.39 | 67.56 | 22.67 | 1.272 | 0.427 |
| Subject16 | Run | 107.78 | 9.74 | 2.03 | 0.18 | 50.17 | 9.03 | 0.945 | 0.170 |
| Subject17 | Sprint | 113.05 | 5.51 | 1.86 | 0.09 | 105.57 | 10.24 | 1.739 | 0.169 |
| Subject17 | Run | 114.05 | 19.92 | 1.88 | 0.33 | 109.43 | 39.21 | 1.803 | 0.646 |
| Subject18 | Sprint | 91.03 | 4.73 | 1.18 | 0.06 | 87.39 | 9.15 | 1.135 | 0.119 |
| Subject18 | Run | 84.10 | 10.21 | 1.09 | 0.13 | 75.29 | 18.30 | 0.978 | 0.238 |
| Subject19 | Sprint | 115.93 | 12.18 | 1.40 | 0.15 | 108.49 | 22.60 | 1.306 | 0.272 |
| Subject19 | Run | 125.14 | 5.69 | 1.51 | 0.07 | 125.56 | 11.47 | 1.511 | 0.138 |
| Subject20 | Sprint | 154.05 | 4.87 | 1.98 | 0.06 | 184.14 | 11.74 | 2.367 | 0.151 |
| Subject20 | Run | 124.75 | 17.64 | 1.60 | 0.23 | 122.29 | 32.74 | 1.572 | 0.421 |
| Subject21 | Sprint | 96.86 | 1.02 | 1.46 | 0.02 | 77.61 | 1.63 | 1.169 | 0.025 |
| Subject21 | Run | 98.59 | 9.31 | 1.48 | 0.14 | 81.04 | 15.09 | 1.220 | 0.227 |
| Subject22 | Sprint | 169.11 | 15.66 | 2.50 | 0.23 | 101.97 | 18.15 | 1.506 | 0.268 |
| Subject22 | Run | 145.79 | 11.41 | 2.15 | 0.17 | 75.61 | 11.54 | 1.117 | 0.170 |
| Subject23 | Sprint | 110.72 | 3.49 | 1.98 | 0.06 | 86.05 | 5.43 | 1.542 | 0.097 |
| Subject23 | Run | 102.51 | 2.55 | 1.84 | 0.05 | 73.76 | 3.69 | 1.322 | 0.066 |
| Subject24 | Sprint | 107.20 | 4.90 | 1.54 | 0.07 | 85.34 | 7.80 | 1.224 | 0.112 |
| Subject24 | Run | 94.26 | 4.97 | 1.35 | 0.07 | 66.02 | 6.80 | 0.947 | 0.098 |

Summary table of subject means and standard deviations (SD) at sprint and running speeds for peak values of tendon stress, mass-specific tendon stress, elastic energy storage, and mass-specific elastic energy storage. All tendon stress and elastic energy storage values are means calculated using the peak instantaneous values for each subject, for each step. An ‘NA’ for the SD means that only one step with useable data was recorded for that speed for that subject.

**References**

1. Scholz, M. N., Bobbert, M. F., Soest, A. J. van, Clark, J. R. & Heerden, J. van. Running biomechanics: shorter heels, better economy. *J Exp Biol* **211**, 3266–3271 (2008).
